# The Role of Depression Screening and Treatment in Achieving the UNAIDS 90–90–90 Goals in Sub-Saharan Africa

**DOI:** 10.1007/s10461-019-02593-7

**Published:** 2019-07-17

**Authors:** Kazione Kulisewa, Melissa A. Stockton, Mina C. Hosseinipour, Bradley N. Gaynes, Steve Mphonda, Michael M. Udedi, Brian W. Pence

**Affiliations:** 1 Department of Mental Health, College of Medicine, University of Malawi, Private Bag 360, Blantyre, Malawi; 2 Epidemiology Department, University of North Carolina at Chapel Hill Gillings School of Global Public Health, 135 Dauer Dr, Chapel Hill, NC 27599 USA; 3 Tidziwe Centre, University of North Carolina Project-Malawi, Private Bag A-104, Lilongwe, Malawi; 4 Department of Psychiatry, University of North Carolina at Chapel Hill School of Medicine, 333 S Columbia St, Chapel Hill, NC 27516 USA; 5 NCDs & Mental Health Unit, Ministry of Health, Malawi, Ministry of Health, Capital City, P. O. Box 30377, Lilongwe 3, Malawi; 6 Department of Medicine, University of North Carolina at Chapel Hill School of Medicine, 321 S Columbia St, Chapel Hill, 27516 North Carolina USA

**Keywords:** Mental health disorders, Depression, Sub-Saharan Africa, HIV/AIDS, UNAIDS 90–90–90

## Abstract

Despite widespread HIV screening and treatment programs across sub-Saharan Africa, many countries are not on course to meet the Joint United Nations Program on HIV/AIDS 90–90–90 targets. As mental health disorders such as depression are prevalent among people living with HIV, investment in understanding and addressing comorbid depression is increasing. This manuscript aims to assess depression and HIV management in sub-Saharan Africa using a 90–90–90 lens through a discussion of: depression and the HIV care continuum; the state of depression screening and treatment; and innovations such as task-shifting strategies for depression management. Due to the lack of mental health infrastructure and human resources, task-shifting approaches that integrate mental health management into existing primary and community health programs are increasingly being piloted and adopted across the region. Greater integration of such mental health care task-shifting into HIV programs will be critical to realizing the 90–90–90 goals and ending the HIV epidemic.

## Introduction

Despite widespread HIV screening and improved treatment programs across sub-Saharan Africa (SSA), many countries are not on course to meet the Joint United Nations Program on HIV/AIDS (UNAIDS) 90–90–90 targets. Mental health problems—particularly depression—remain a prevalent comorbidity in people living with HIV (PLHIV), directly impacting their quality of life and hampering each 90–90–90 goal from diagnosis to enrollment and retention in treatment, and ultimately viral suppression. This suggests HIV treatment programs in SSA will have to embrace innovations that address mental health challenges.

This manuscript aims to assess depression and HIV management using a 90–90–90 lens due to the high prevalence of depression [1] and its importance as a public health burden [2, 3]. First, we will provide some background on HIV and mental health care in SSA. Next, we will discuss depression as it relates to the HIV care continuum, with a particular focus on the 90–90–90 targets. We then will provide an overview of the state of depression screening and management in SSA. Finally, we will describe task-shifting strategies for managing depression among PLHIV and highlight an example of one such program that is integrating depression screening and treatment into HIV care initiation in Lilongwe, Malawi.

## Background

### Burden of HIV in SSA

Of the 36.7 million PLHIV globally in 2015 [4], an estimated 25.5 million resided in the sub-Saharan region [4, 5]. This region still accounted for 66% of the 2.1 million new infections and 800,000 of the 1.1 million HIV-related deaths registered that year [4]. As in other countries, up to 40% of HIV-positive individuals were unaware of their HIV status [6], and of those confirmed to be living with HIV, less than half were on antiretroviral treatment (ART) [4].

These figures demonstrate progress in the fight against HIV, especially in light of the reduced mortality rates, averaging a 39% reduction in HIV-related mortality between 2005 and 2013 [5, 7]. However, the high morbidity of the epidemic continues to burden sub-Saharan communities, hampering the economic productivity of some of the world’s least resourced and low-income countries [6].

UNAIDS introduced the 90–90–90 goals in 2013 to help guide efforts to end the HIV epidemic by 2030. These targets aim to ensure 90% of PLHIV know their status, 90% of those diagnosed with HIV receive sustained ART, and 90% of individuals on ART are virally suppressed by 2020 [8]. To achieve these goals, health systems need to adopt innovations to tackle barriers that have hitherto been under-recognized or unaddressed. The role and impact of co-morbid mental health disorders in HIV treatment is one such area that is increasingly being recognized [9].

### Mental Health Disorders and HIV

Mental health disorders are diverse conditions that present with abnormalities of thought, emotion and behavior, frequently impairing the function of the individual [10]. Mental health disorders and HIV frequently co-exist. Various studies from African countries have estimated that the prevalence of mental health problems in PLHIV range from 19% [11] to about 50% [1]. One aspect of the complex relationship between mental disorders and HIV is that individuals with mental health or substance use disorders are more likely to engage in behaviors that may increase their risk of acquiring HIV and transmitting the virus, and are less likely to engage with healthcare providers [12].

Improvements in HIV care, namely widespread programs which encourage proactive and provider-initiated HIV testing as well as early and immediate initiation of ART, can potentially render HIV a chronic and manageable disease. However, as seen with other chronic medical conditions, mental health disorders are common, with the lifetime prevalence of any mental health disorder in HIV-positive populations estimated to range from 38 to 75% [12, 13]. While psychiatric conditions can occur at any stage of HIV infection, they tend to be more prevalent with HIV progression and end-stage disease [12, 14].

The considerable overlap in symptomatology between HIV and the somatic symptoms that feature in common mental health disorders complicates the picture, with HIV care providers frequently under-recognizing the potential burden of comorbid mental health disorders in PLHIV [12]. Unrecognized and untreated, mental health disorders have the potential to impact the entire HIV care continuum from HIV prevention strategies, to diagnosis and retention in ART programs. Although potentially any mental health disorder may have an influential association with HIV, the World Health Organization (WHO) emphasizes the importance of neurocognitive disorders, substance use disorders and depression [15]. The focus here will be on depression, as it has the highest burden of disease globally and in SSA [16].

### Depression

Depression is very common, with a lifetime prevalence in the global general population estimated at 15–22% [12, 17]. Among individuals with chronic illness such as HIV, depression is even more common [12, 18], estimated to be two to three times more prevalent [19].Various global studies have reported the prevalence of depressive disorders and symptoms in PLHIV to range from 9 to 45%, the variance largely attributed to different diagnostic or screening tools [12, 19, 20].

Poverty and stressful life events have well recognized associations with depression. High rates of these same factors are also observed in PLHIV [19, 21]. However, the relationship between HIV and depression goes beyond common risk factors. Evidence shows that depression can precede infection and is associated with risk factors for HIV acquisition [18]. Further, depression can directly impact HIV clinical progression and is associated with higher morbidity and mortality [21]. Reciprocally, discrimination and stigma remain prevalent among PLHIV, HIV-related stigma when internalized makes depression a likely outcome [22, 23]. The chronic debilitating nature of HIV infection in itself can precipitate depression [18]. Unsurprisingly, depression is the most common psychiatric comorbidity in PLHIV.

### Innovations in Tackling the HIV Epidemic

Conventional approaches to HIV management have involved counseling adherence, monitoring viral loads and CD4 counts, and a focus on treating opportunistic infections. A mounting evidence base suggests that mental health monitoring is equally important to achieving optimal HIV care outcomes [9, 21].

## Depression and HIV in SSA: Prevalence, Associations, Challenges and Opportunities

### Prevalence of Depression

The body of regional literature exploring the relationship between comorbid depression and HIV is a growing one. Several studies conducted in SSA have demonstrated that depression is pervasive among PLHIV. A recent systematic review reported the prevalence of major depressive disorder to range between 13 and 24% and subthreshold depressive symptoms to range between 9 and 32% [19]. Similar prevalences have been reported by other sub-Saharan studies [24, 25].

### Depression and HIV Testing (‘First 90’)

To date, few studies have investigated the influence of depression on HIV testing. One study conducted in South Africa found that patients with severe depression had higher odds of late testing, defined as presenting within 3 months of diagnosis with a CD4 count ≤ 200, as compared to those without depression [26]. Outside of SSA, a study conducted in Nepal found an association between non-utilization of HIV testing and counseling and depression among female sex workers [27]. Some studies suggest that depression is associated with reduced or delayed mental health care-seeking behavior [28–30]. Any reduction in care-seeking behaviors could potentially have an impact on HIV testing rates in settings such as SSA that utilize every interaction with the health system as an opportunity to refer patients for HIV testing. While research is needed to better understand depression at the community level as it relates to HIV testing, there is some evidence to suggest depression may ultimately undermine HIV testing and the ‘First 90.’

### Depression, Linkage to ART, and ART Adherence (‘Second 90’)

Several studies have demonstrated that depressed individuals are less likely to be linked to ART care or start treatment [26, 31–33]. One study conducted in South Africa found that depressed individuals had higher odds of waiting at least 3 months following an HIV diagnosis to initiate ART compared to their non-depressed counterparts [26]. Another study conducted in South Africa found individuals with depressive symptoms, who were referred for HIV testing by a healthcare provider, were less likely to obtain a CD4 count—the first step in initiating care—than those with no depressive symptoms who self-referred for testing [32]. Some research has hypothesized that this trend is due to depressed individuals being unprepared or unable to cope with their diagnosis [34].

Depression has consistently been shown to have a significant association with poor adherence to ART [20, 35]. While the reasons for this association are not fully clear, the loss of interest, poor concentration, poor motivation and suicidality—which are characteristic of depression—are all plausible factors which can impair adherence [17, 20].

Unlike most medications which require adherence levels exceeding 80% to achieve the desired clinical effect, ART requires much higher adherence levels (ideally exceeding 95%), and non-adherence increases the risk of treatment failure and viral resistance [12, 36, 37]. The chronicity of HIV means that this needs to be sustained lifelong adherence. A systematic review of SSA literature established that PLHIV with untreated depression were 55% less likely to be adherent to ART as compared to their peers not suffering from depression [25].

Successful management of depression can improve ART adherence; several studies have reported that a decrease in depressive symptoms was associated with improved ART adherence [2, 12, 35, 38]. Untreated depression may hamper early linkage to care and ART adherence thereby impede achievement of the ‘Second 90’ target.

### Depression, Viral Suppression (‘Third 90’), Clinical Outcomes and Mortality

HIV clinical outcomes improve with the treatment of depression. One of the few studies conducted in SSA exploring the effect of depression management among PLHIV reported improved CD4 counts, viral suppression, and a four-fold improvement in the proportion reporting good health in a cohort of Cameroonian HIV-positive patients who received 4 months of antidepressant treatment [2].

In the pre-ART era, studies in high-income countries showed that men who have sex with men (MSM) living with HIV showed faster HIV progression if they had comorbid depression. The risk of AIDS-related mortality was particularly high in the presence of prominent depressive somatic symptoms [21]. These findings are suggestive that the impact of depression on HIV progression is mediated through more than impaired adherence to ART.

Despite profound improvements in mortality due to increased access and earlier initiation of ART currently, PLHIV with comorbid depression remain at a two-fold higher risk of death from AIDS-associated causes as compared to their non-depressed counterparts [21, 39–42]. Conversely, PLHIV who demonstrate positive affect or who have been managed for depression are known to be less likely to die from AIDS complications [21]. Untreated depression likely has a negative effect on immunological response and viral suppression, being associated with greater declines in CD4 counts and increases in viral load [21].

Depression appears to be a significant barrier to sustained engagement in HIV care [40, 43]. The extensive literature on the relationship between depression and ART adherence emphasizes that the ‘Second 90’ and ‘Third 90’ targets—which address retention in care, sustained ART and viral suppression [8]—are unlikely to be achieved without treating depression.

## Integrating Mental Health Management into HIV care in SSA

### Depression Screening Among PLHIV in SSA

Various tools have been developed or adapted and validated in SSA for the screening of depression in various populations. Of particular relevance are the screening tools that have been shown to have utility among PLHIV.

In Malawi and Zimbabwe, vernacular versions of the Edinburgh Postnatal Depression Scale (EPDS) have been validated and are administered by healthcare providers in light of low patient literacy [44, 45]. Use of the EPDS is increasingly being advocated in routine antenatal and postnatal care in areas of high HIV prevalence [45].

The Patient Health Questionnaire-9 (PHQ-9) has shown value as a screening tool in a variety of global settings. Importantly, it has been successfully adapted and validated in diverse sub-Saharan settings among PLHIV. Although it has demonstrated variable sensitivity, the PHQ-9 has shown acceptable specificity and acceptable ease of use by non-specialized primary healthcare workers [46, 47].

Although much of the research around depression and HIV has targeted adults while ignoring the high prevalence of HIV among adolescents in SSA, a vernacular version of the Beck Depression Inventory-II has been developed for use in Malawian children and adolescents [48].

### Depression Management

A 2011 systematic review of depression interventions found that psychological interventions, especially cognitive-behavioral therapy, were particularly effective in treating comorbid depression in PLHIV, while psychotropic medication had a somewhat less impressive evidence base [18]. However, a separate meta-analysis supports the efficacy of antidepressants for HIV-positive individuals [49].

Strikingly, 90% of the literature was from high-income North American and European countries, and a significant proportion of the studies (35%) had been conducted with MSM or bisexual populations [18]. SSA, despite having much of the global burden of HIV, is disproportionately under-represented in the literature. Furthermore, the overwhelming modes of HIV transmission in Africa are heterosexual or mother-to-child-transmission. Arguably, Western evidence-based interventions may not demonstrate similar efficacy when adopted in sub-Saharan populations.

While not an exhaustive list of the interventions developed for management of depression regionally, we will describe some interventions that have shown efficacy in sub-Saharan populations living with HIV.

‘The Friendship Bench’ is a psychosocial innovation based on problem-solving therapy that was developed in Zimbabwe. A low-cost intervention that is delivered by lay individuals, including grandmothers—in what may be viewed as a reprising of their familiar cultural roles as advisors—the Friendship Bench has a good evidence base for its efficacy in managing common mental health disorders and depression symptoms in both HIV-uninfected populations and PLHIV [50]. It has particular appeal in sub-Saharan settings because of its cultural relevance and low cost, suggesting positive implications for sustainability [50, 51].

Similarly, in Tanzania, a 6-week intervention using group counseling with elements of problem-solving therapy in antenatal women living with HIV has shown modest efficacy in reducing depressive symptoms as compared to the usual standard of care pre- and post-HIV test counseling [52]. Group counseling has also been piloted in South Africa, where lay HIV counselors trained in an adapted interpersonal therapy intervention have facilitated group therapy of demographically heterogeneous adult PLHIV over eight sessions to effectively reduce symptoms of depression [3].

Other group work with PLHIV and comorbid depression has occurred in Uganda with the development of group support psychotherapy (GSP). This is a combined approach that, amongst other elements, integrates theories from cognitive-behavioral therapy and social learning to address low income, deficits of social support and coping skills which may be perpetuating factors in depressed PLHIV. The effects of GSP can be sustained, with treated individuals exhibiting improved functionality and lower depressive scores 6 months after the last session of the intervention [53].

Although depression directly impacts ART adherence, the evidence base suggests that simply treating depression in isolation doesn’t necessarily improve adherence [54]. Accordingly, some regional interventions have used approaches that target both adherence and depression symptoms. Life-Steps is a brief single session intervention that improves ART adherence primarily through cognitive-behavioral techniques including motivational interviewing, psychoeducation and problem-solving strategies to overcome barriers to ART adherence. *Nzira Itsva* (Shona for ‘New Direction’) is an innovation adapted from Life-Steps that is delivered by lay counselors, consisting of a single session which can be augmented with subsequent ‘booster’ sessions that has been trialed in Zimbabwe [55]. While the efficacy of Nzira Itsva in improving adherence is yet to be established, ART clients have found the program acceptable.

Building on Nzira Itsva and the Friendship Bench, a Zimbabwean trial combined both interventions to produce a stepped care model. ART counselors administered an initial session of problem-solving therapy specifically targeting poor adherence and subsequently followed this up with three depression-specific problem-solving therapy sessions. ART counselors referred patients with inadequate remission of depression symptoms after four sessions to a clinical psychologist who provided a further two sessions. Patients who manifested with significant symptomatology after a 6-week intervention were eligible for referral to a psychiatrist and possible antidepressant initiation [54].

An alternative South African approach to addressing adherence and depressive symptoms is *Ziphamandla* (meaning ‘Empower Yourself’). Structured, manualized cognitive behavioral therapy is delivered by nurse practitioners under weekly supervision from mental health specialists. Using this approach, patients have demonstrated sustained ART adherence and improvement in depressive symptoms [56].

Measurement-based care (MBC) is an evidence-based algorithm in which non-psychiatric primary healthcare workers prescribe and manage antidepressants. Decision-making is guided by two criteria: depression symptom level—as assessed by a standardized tool—and side effect presence and severity. Clinical success using these task-shifting methods has been reported in several American trials and a pilot in Cameroon showed good clinical outcomes in PLHIV, with improved immunological and virological response after 4 months of MBC [2]. MBC has also been successfully piloted in Tanzania [57] and evaluated in a 10-site clinic-randomized trial in Uganda [58].

### Task-shifting Approaches to Combat Deficits in Mental Health Resources

As emphasized above, mental health disorders contribute significantly to the burden of disease regardless of HIV status in SSA, yet the region has little mental health infrastructure and human resource capacity to address this burden [2, 59–61]. In a region with 0.05 psychiatrists per 100,000 population, it is estimated that 75% of the individuals requiring mental health interventions are not accessing them [2].

An approach to this disparity recommended by the WHO is the integration of mental health services into existing primary and community health services [61]. Improving the capacity of general clinicians [62] and primary health and lay health workers to effectively screen and manage common mental health disorders is a practical and cost-effective method of scaling up mental health services and tackling the vast treatment gap. Bringing mental health to the community further serves to address the discrimination and stigma that have traditionally been associated with psychiatric disorders [61].

In Malawi, such approaches have been piloted and have led to improved mental health knowledge and perceived self-efficacy in managing mental health disorders in community lay health workers [61]. Similarly, in Zimbabwe and South Africa, lay healthcare workers provide psychosocial interventions to PLHIV, which have been shown to be effective in reducing symptoms of common mental health disorders [3, 51]. Task-shifting to trained primary healthcare workers also allowed successful delivery of antidepressants to PLHIV in Cameroon [2].

## A Project SOAR Case Study: Integrating Depression Screening and Management into HIV Care in Malawi

Successful HIV programs in Malawi have now reduced the adult HIV prevalence to 9.2% as of 2016. As compared to 2010 data, new HIV infections and deaths have declined by 46% and 39%, respectively [63]. However, HIV testing, ART adherence and retention in care remain problematic with estimates suggesting that among the country’s one million individuals currently living with HIV, only 66% are on sustained ART and 59% have achieved viral suppression [63]. Addressing and managing the mental health conditions prevalent among those who do access HIV testing may be key to attaining the UNAIDS-90–90–90 goals.

Supported by Project SOAR, a funding portfolio from the United States President’s Emergency Plan for AIDS Relief (PEPFAR) and the United States Agency for International Development (USAID), we are evaluating Malawi’s first program that integrates depression screening and management into HIV care initiation. The project addresses the paucity of mental health and HIV literature in Malawi by establishing the prevalence of depression among patients newly initiating ART, assesses how integration of ART and depression care services can be implemented, improves the capacity of primary health workers in screening and managing depression, and evaluates the impact of integrated HIV and depression services on HIV clinical outcomes (including appointment attendance and viral suppression). The project utilizes depression management interventions that have been proven to be effective in the region.

In line with screening approaches used in other SSA countries, we adapted the PHQ-9 to screen and monitor depression [46, 64]. A Chichewa (the vernacular of the central region of Malawi) version of the PHQ-9 first underwent a process of cultural validation for use among patients with diabetes [65]. We further refined this tool for use in HIV care clinics through an iterative process that involved observation of clinical PHQ-9 administration and qualitative assessment to arrive at a screening tool that adequately captures the depressive symptoms, yet is culturally understandable [66]. HIV testing counselors administer the first two questions of the PHQ-9, known as the PHQ-2, which screen for the core symptoms of depressed mood or anhedonia, during HIV post-test counseling. ART providers (clinicians and nurses) administer the remaining seven questions of the PQH-9 during ART initiation to patients who endorsed at least one of the PHQ-2 questions. The adapted tool considers PHQ-9 scores of 5–9 as indicative of mild depression and scores of ≥ 10 as moderate to severe depression. Since March 2017, the two pilot health centers in the district of Lilongwe have consistently used the Chichewa language PHQ-9 to screen patients initiating ART for depression.

As part of this program, we trained healthcare providers to manage depressed PLHIV through either psychosocial interventions or antidepressant medication. Through regional collaboration with ‘The Friendship Bench’ developers, we trained lay community healthcare workers to provide problem-solving therapy to patients with mild depression. ‘The Friendship Bench’ in the Malawian context consists of six one-on-one sessions lasting 45–60 min and held monthly on clinic premises with community health workers over a period of 6 months. We trained ART providers to use MBC to manage moderate to severe depression with a prescription algorithm that uses PHQ-9 scores (to monitor depressive symptoms) and side effect severity to guide antidepressant prescription and adjustment. Providers prescribe sustainable, freely available antidepressants (amitriptyline or fluoxetine) that are included on the country’s list of essential drugs [67].

Preliminary results have demonstrated successful adoption of the screening tool, with 92% of 2414 newly diagnosed PLHIV completing the initial PHQ-2 screen (n = 2216) between April 2017 and November 2018. Among the 2080 patients who were enrolled in the program evaluation and completed the initial PHQ-2 screen, 24% (n = 502) had mild to severe depression and 6% (n = 131) had moderate to severe depression. Although not formally evaluated, the knowledge and attitudes of primary healthcare workers toward mental health disorders appear to have positively changed as their capacity to screen and recognize depression has increased. From an implementation perspective, the integration of depression screening within HIV care clinics appears a feasible and acceptable innovation as reflected by the proportion of PLHIV who are completing the screening tool with minimal disruption of workload and clinic flow. Currently the program is evaluating the clinical outcomes of the intervention with respect to retention in HIV care, viral suppression, and depression remission.

## Conclusion

As mental health disorders such as depression are highly prevalent in PLHIV, the role and impact of comorbid mental health disorders in the HIV continuum of care is an increasingly recognized area which has the potential to positively influence the response to HIV. Although limited research has focused on the impact of current mental disorders on HIV testing (‘First 90’), the literature suggests that comorbid depression plays a pivotal role in reducing engagement with health services, influencing early loss to care and poor adherence among patients receiving ART (‘Second 90’). There is also compelling evidence that depression has an adverse effect on viral suppression (‘Third 90’) and immunity leading to greater rates of HIV-related mortality. The UNAIDS 90–90–90 goals may not be achieved without the necessary focus and resource allocation to address the depressive disorders that frequently co-exist in PLHIV.

Sub-Saharan Africa, which accounts for the greatest burden of HIV-related disease globally, also suffers from a lack of mental health infrastructure and human resource to tackle comorbid mental health disorders in PLHIV. To address this disparity, task-shifting approaches that aim to integrate mental health management into existing primary and community health programs are increasingly being piloted and adopted across the region. Greater integration of such task-shifting mental health care approaches into ongoing HIV care will be critical to fully realize the UNAIDS 90–90–90 goals and end the HIV epidemic. As mental health disorders remain highly stigmatized in SSA, integration of mental health care also represents a unique opportunity to educate health care providers about mental health and combat negative attitudes.
